# Participatory hackathon to determine ecological relevant endpoints for a neurotoxin to aquatic and benthic invertebrates

**DOI:** 10.1007/s11356-024-32566-w

**Published:** 2024-02-28

**Authors:** Sofie B. Rasmussen, Thijs Bosker, Giovani G. Ramanand, Martina G. Vijver

**Affiliations:** 1 Institute of Environmental Sciences, Leiden University, P.O. Box 9518, 2300 RA Leiden, The Netherlands; 2 Leiden University College, Leiden University, P.O. Box 13228, 2501 EE The Hague, The Netherlands

**Keywords:** Behavioural endpoints, Ecotoxicity, Education, Sulfoxaflor, Non-conventional endpoints, Biomechanistic endpoints

## Abstract

The aim of this study is twofold: i) to determine innovative yet sensitive endpoints for sulfoxaflor and ii) to develop best practices for innovative teaching in ecotoxicology. To this end, a group of 52 MSc students participated in an environmental *hackathon*, during which they did creative toxicity testing on 5 freshwater invertebrate species: *Daphnia magna, Chironomus riparius, Asellus aquaticus, Lymnaea stagnalis,* and *Anisus vortex*. Involving the students in an active learning environment stimulated increased creativity and productivity. In total, 28 endpoints were investigated, including standard endpoints (e.g., mortality) as well as biomechanistic and energy-related endpoints. Despite high variances in the results, likely linked to the limited lab experience of the students and interpersonal differences, a promising set of endpoints was selected for further investigation. A more targeted follow-up experiment focused on the most promising organism and set of endpoints: biomechanistic endpoints of *C. riparius* larvae. Larvae were exposed to a range of sulfoxaflor concentrations (0.90–67.2 μg/L) for 21 days. Video tracking showed that undulation and swimming were significantly reduced at 11.1 μg sulfoxaflor/L after 9 days of exposure, and an EC_50_ = 10.6 μg/L for mean velocities of the larvae in the water phase was found. Biomechanistic endpoints proved much more sensitive than mortality, for which an LC_50_ value of 116 μg/L was found on Day 9. Our results show that performing a hackathon with students has excellent potential to find sensitive endpoints that can subsequently be verified using more targeted and professional follow-up experiments. Furthermore, utilising hackathon events in teaching can increase students’ enthusiasm about ecotoxicology, driving better learning experiences.

**Supplementary Information:**

The online version contains supplementary material available at 10.1007/s11356-024-32566-w.

## Introduction

Considering the increasing number of environmental contaminants with neurotoxic potential (Busch et al. 2016), there is an urgent need for novel and sensitive endpoints to screen chemicals for potential neurotoxic effects (Morrissey et al. 2015; Legradi et al. 2018). Currently, risk assessments largely focus on mortality, growth, and reproduction (Schuijt et al. 2021). However, studies have shown that focusing solely on these endpoints might underestimate the potential toxicity of contaminants with neurotoxic potential to aquatic invertebrates (for a review on this, see Sarma and Nandini 2006). This is illustrated by research on neonicotinoids; Raby et al. (2018) reported a difference in sensitivity of up to 3 orders of magnitude when comparing mobility of mayflies to mortality resulting from 6 different neonicotinoids. The same observation is reported by Barmentlo et al. (2019), with effects on swimming abilities of mayfly nymphs happening at concentrations as low as 0.84 μg/L thiacloprid, while no mortality was observed even at more than tenfold higher concentrations (Barmentlo et al. 2019). While behavioural endpoints show promising results in predicting sensitivity, the ecological relevance cannot be ignored (Ågerstrand et al. 2020; Bownik and Wlodkowic 2021). Individual traits such as behaviour can have important consequences on species population dynamics through changes in feeding or predator-prey interaction (Weis et al. 2001; Relyea and Hoverman 2006). Impaired mobility has been found to leave aquatic organisms vulnerable to predators and directly affect the survival rates (Langer-Jaesrich et al. 2010; Gutierrez et al. 2013; Moore et al. 2019). Some examples of individual behaviour and their link to population-level impacts are summarised in Ågerstrand et al. (2020). For instance, avoidance behaviour in European eel (*Anguilla anguilla*) was linked to population-level effects and has been used as a key study for the regulatory decision on methyl tertiary-butyl ether within the European Union Risk Assessment Report.

Sulfoxaflor has recently received significant attention as an alternative to the controversial neonicotinoid pesticides (Siviter et al. 2018; Azpiazu et al. 2021). Sulfoxaflor is a relatively new insecticide in the group of sulfoxamines, whose primary site of action being nerve action (Gauthier and Mabury 2021; IRAC 2023). Similar to neonicotinoids, sulfoxaflor is considered a nicotinic acetylcholine receptor (nAChR) competitive modulator (Cutler et al. 2012; Watson et al. 2017). As the mode of action (MoA) is similar for the group of sulfoxamines and neonicotinoids, there are concerns about similar adverse effects on the environment (Siviter et al. 2018; Atta et al. 2021). Currently, the EU concludes that the risk to aquatic life is low, based on standard Tier 1 risk assessment (EFSA 2014; EFSA 2020).

However, recent studies show that the toxicity of sulfoxaflor on other aquatic species might be higher than first assumed. For example, Maloney et al. (2020) estimate an acute LC_50_ value of 164.4 μg/L for *Chironomus dilutus*, more than 600 times lower than the US Environmental Protection Agency (USEPA) recommended benchmark for aquatic invertebrates of > 100,000 μg/L (US Environmental Protection Agency 2018). Similar acute LC_50_ values of 84.1 μg/L were found for *Chironomus kiinensis* by Liu et al. (2021). Noteworthy, the same study found chronic sublethal effects on growth and emergence at 20 μg/L (Liu et al. 2021), around 2 times lower than the no observable effect concentration (NOEC) of 38.4 μg/L for *Chironomus riparius* considered for the EU risk assessment (EFSA, 2014). This large difference between the sensitivities highlights the urgent need for testing across multiple endpoints to understand better the full potential risks of sulfoxaflor to aquatic ecosystems.

As for other potentially neurotoxic chemicals, there is an urgent need for data beyond standard endpoints to quickly and efficiently determine effects on aquatic populations (Taylor and Scroggins 2013). However, finding potential novel endpoints can be time-consuming and costly. One option to solve this is participatory research, which has the potential for broad screening and effective data collection (Strasser et al. 2019). Participatory research originated in healthcare but has lately transferred into other disciplines, including sustainability questions (Lang et al. 2012) and teaching situations (Areljung et al. 2021). Its foundation is that researchers receive help from volunteering participants, such as students or the public (Gibson et al. 2017). The researcher gets new input on the research topic and a large amount of data (Strasser et al. 2019). In return, the participants get educated within the field (Cornwall and Jewkes 1995) and can, in other cases, be part of decision-making processes (Jull et al. 2017). Another benefit of participatory research, and the focus of this study, is collaborating with participants to increase collective creativity in a hackathon event inspired by commercial computing hacking held in software development (Cwikel and Simhi 2021). Hackathons are time-limited events where participants in groups produce innovative solutions to selected issues (Angarita and Nolte 2020); here, the search is for sensitive endpoints to non-target organisms.

The objective of this study was twofold. First, the goal was to determine whether a hackathon event with MSc students from Leiden University could be used to detect potentially sensitive aquatic species and endpoints when exposed to sulfoxaflor and to develop best practices and methods to get fast and large amounts of reliable data on potential sublethal effects induced by neurotoxic chemicals. Secondly, based on the data collected in the Hackathon, a subset of endpoints were tested by researchers to validate the results and to determine more accurate dose-response relationships.

To test a broad range of aquatic species, 5 species were included in this study: *Daphnia magna*, *Chironomus riparius*, *Asellus aquaticus*, *Lymnaea stagnalis*, and *Anisus vortex*. The species were selected based on earlier shown sensitivities to sulfoxaflor or similar compounds such as the neonicotinoids. Augusiak and Van den Brink (2016) showed behavioural changes in *A. aquaticus* when exposed to chlorpyrifos and imidacloprid, with both pesticides altering step length of the organisms in concentrations at 0.6 and 37.5 μg/L, respectively. Furthermore, Vehovszky et al. (2015) showed that Thiacloprid can inhibit cholinergic neurotransmission by blocking 90 % of excitatory postsynaptic potentials in *L. stagnalis*. Additionally, Prosser et al. (2016) describe neonicotinoids-induced effects on growth of two mollusc species: *Lampsilis Fasciola* and *Planorbella pilsbryi.* Over the 28 days of study, growth and biomass production were considerably more sensitive than mortality for *P*. *pilsbryi* (Prosser et al. 2016). *D. magna* and *C. riparius* were selected as standard species for comparison with known acute sensitivities (Dow 2014; Maloney et al. 2020).

## Methods

### Part 1: hackathon in ecotoxicity testing

The hackathon was performed in January 2022 over a period of four weeks, with a total of 52 MSc students participating in a regular course in ecotoxicology at Leiden University, the Netherlands. Within the assignment, students were stimulated to think beyond using conventional endpoints and to create innovative testing setups. The focus was on organism-fitness or population-relevant endpoints that can capture neurotoxic responses for non-target species in the aquatic system (both in the water column and the sediment). The follow-up experiment (“Part 2: follow-up experiment”) made use of the knowledge gained in the Hackathon with few modifications for optimisations related to robustness (e.g., optimal sample size based and diminishing uncertainties due to methodological errors). The selection criteria for the follow-up experiment were ecological relevance of the endpoints, sensitivity of the endpoints, and sensitivity of the organism. The students were divided into 8 groups and tested a total of 12 different endpoints for 5 different aquatic invertebrate species. The results gave a broad overview of responses (28 organism–endpoint combinations).

#### Hackathon timeline

Within the constraints of the four-week course, the students were introduced to the topic in the first week, whereafter, an experimental part was conducted in the second and third weeks of the course. The four weeks were finalised with a presentation from each group on the obtained results and knowledge and a report following scientific writing criteria. Table 1 describes the itinerary of the Hackathon, showing a mix of passive teaching with lectures in a classroom and group work in a laboratory setting. For the group work and experimental part, at least one supervisor was present at all times.

**Table 1:** Itinerary followed in the Hackathon. Each week was equivalent to a normal workload of 40 h per week. The students were expected to be present at least 90% of the time. Lectures were performed in a classroom with the teacher presenting for a passive audience, whereas group work and lab work were performed in larger teaching facilities with laboratory equipment present

Week	Teaching methods	Content
1	Lectures, group work, and presentations	Introduction to hackathon setup Lectures on key aspects of ecotoxicity and risk assessment Lectures on experimental design and statistical analyses Group work with the design of the research question Presentation on individual research questions and methodology
2	Experimental lab work and lectures	Conduction of the experiment Lectures on effect modelling Lectures including case studies from real-life research
3	Experimental lab work and lectures	Continue conduction of the experiment Lectures including case studies from real-life research
4	Presentations and writing	Presentations on obtained results Hand-in of a report based on obtained results and a broader perspective
5	Evaluation	An in-class discussion on improvements to the course took place Furthermore, students were able to give anonymous feedback in the course evaluation

#### Test species

The species used were *D. magna, C. riparius, A. aquaticus, L. stagnalis,* and *A. vortex*. Eggs of *C. riparius* were hatched under test conditions less than two days before initiating the experiment. They were kindly provided from a long-lasting culture of the University of Amsterdam. *D. magna* were taken from an in-house culture at Leiden University and maintained according to the conditions prescribed in OECD Test Guideline 211 (OECD 2012). Newly hatched neonates (< 24 h old) were used. *L. stagnalis* and *A. vortex* were collected from an artificial pond about 1 month prior to the start of the experiments (Leiden, Netherlands). Before the experiment, both species were kept under experimental conditions in Elendt M7 medium (OECD 2012) and fed organic lettuce *ad libitum*. *A. aquaticus* were collected from a nearby pond (outdoor test facility Living Lab Leiden; Netherlands) about 6 months prior to the experiment and kept in an outdoor aquarium filled with pond water and fed DECOTABs *ad libitum* (details can be found in *supplementary information*).

#### Exposure setup

In this study, the 5 test species were exposed to sulfoxaflor (97.8%; CAS No. 946578-00-3; AccuStandard, New Haven, USA) for 9 days following the specific details in Table 2. Concentrations of sulfoxaflor were similar for all species at 6 different nominal concentrations: 6.25, 12.5, 25, 50, and 80 μg/L (selected based on Liu et al. 2021, Maloney et al. 2020, Hoffman 2020, and DOW 2014). The number of replicates was between 4 and 15, depending on species and availability of test organisms (Table 2). Experimental design varied slightly between each species to accommodate biological differences. All details can be found in Table 2. Furthermore, all experiments were performed at room temperature (20 ± 1 °C) and natural light (8:16—light to dark cycle). The treatments were allocated at random for each test species. All test vessels contained a minimum of 2–3 cm of overlying water. The medium (Elendt M7 medium; OECD 2012) was replaced with fresh medium on Day 4, except with *D. magna,* which was refreshed every second day due to unwanted algae growth. The pH and dissolved oxygen were measured multiple times (Hach HQ40d multimeter, Hach Ltd., Colorado, USA). Aeration was added after Day 1 for *A. aquaticus* to keep conditions at optimal levels. For tests using *C. riparius* larvae, inorganic sand (grain size of 0.01–2 mm; depth 1–1.5 cm) was added.

**Table 2:** Summary of tested species and associated test conditions used for toxicity testing of sublethal effects of sulfoxaflor in a science Hackathon. All test species were checked for mortality daily

Species	Insect larva *C. riparius*	Isopod *A. aquaticus*	Snails *L. stagnalis* and *A. vortex*	Waterflea *D. magna*
Origin	University of Amsterdam laboratory culture	Field-collected	Field-collected	In-house culture
Life stage	< 48 h old	Adults	Adults	< 24 h old
Exposure concentrations	12.5–50 μg/L	12.5–80 μg/L	12.5–50 μg/L	6.25–12.5 μg/L
Exposure duration	9–10 days	9 days	10 days	9 days
Test vessel	175-mL test beakers	100-mL test beakers	400-mL test beakers	4-mL test beakers
No. of replicates	5–7	4–6	*L. stagnalis*: 6–14 *A. vortex*: 7	7–15
No. of animals/replicate	1–3	2–3	*L. stagnalis*: 1–2 *A. vortex*: 1	1
Feeding during test	Trouvit/tetraphyl 20:1 ratio (0.5 mg/organisms/day	Stinging nettle DECOTABS	Lettuce (4.4 cm^2^/organisms/day)	0.5 mL *Pseudokirchneriella subcapitata* (24 × 10^6^ chlorophyll cells/mL)
Tested biomechanical endpoints	Agitation/undulation Velocity Travelled distance Digging behaviour	Turnover rate Wall bumping Travelled distance Velocity Hiding behaviour	Retraction Movement	Horizontal swimming distance
Energy-related endpoints	Growth (length) Head width	Growth (length)	Growth (weight) Shell size	Growth (length) Spine length

For all species, mortality was recorded daily. Furthermore, students had to measure additional biomechanistic or energy-related endpoints, which they selected based on the literature. Depending on species, growth was recorded on Day 9 as the difference in either wet weight or length. Additionally, each instar stage was determined in *C. riparius* larvae as head width following Watts and Pascoe (2000), and increased spine in the presence of kairomones was assessed for *D. magna* following Rabus and Laforsch (2011). Furthermore, biomechanistic endpoints were recorded on Day 9 (“Behavioural analysis”).

#### Behavioural analysis

For the behavioural analysis of each species, the different groups invented various methods based on the biology of the species. The students were encouraged to think of innovative and unique measures taken scientific literature on the compound and each species into account. Before setting up the experiments, each group performed behavioural trials with culture controls for a better understanding of their ideas. For *D. magna*, swimming abilities were tested using a blacked-out glass aquarium (length = 25 cm) filled with demineralised water. A light source was placed on one end of the aquarium to assess changes in phototactic behaviour (van Gool 1997). Per treatment, 7 *D. magna* were randomly selected and placed opposite the light source. After 30 s, the location of the *D. magna* was noted. The position was scored from 0 to 3, 0 being the start position and 3 being the closest to the light.

Locomotion and reaction were tested for the snails *L. stagnalis* and *A. vortex*. For the locomotion assessment, each snail was placed in a Petri dish, and the distance travelled was measured in 2 min. The reaction was measured as retraction of the snail into the shell after physical stimuli. Depending on the student group running the experiment, this was measured as a retraction or no retraction (*L. stagnalis*) or time from physical touch until response of the snail (*L. stagnalis* and *A. vortex*).

*C. riparius* activity was estimated as either travelled distance over a fixed duration of time or the agitation state of larvae. Each larva was placed in a Petri dish, and the video was recorded for 20–30 s. Recordings were analysed in Tracker (Douglas Brown; version 6.0.6) to determine total travelled distance and velocity. However, only selected frames were used for tracking (1:20). Additionally, the number of frames in which each organism moved (*v* > 0.1 cm s^-1^) was counted to estimate changes in the swimming abilities. Another group based the agitational stage of the larvae by visual inspection, counting the number of performed undulations.

The locomotive behaviour of *A. asellus* was detected in several ways. Mobility was measured by placing 3 organisms from each replicate in a Petri dish to estimate the distance travelled and mean velocity for a duration of 2.5 min. In addition, physical contact among individuals was noted down, as well as time spent near the rim of the Petri dish. Furthermore, reaction rate was noted by placing each individual on its back and measuring the time it took to turn around. Furthermore, locomotive behaviour was determined in a walking channel (length = 22.5 cm) constructed from inorganic sand and demineralised water. The organisms were placed pairwise at one end of the channel, separated from the main body of the channel by a plastic division. The opposite corner was darkened with brown foam paper and had pebbles. The animals were stimulated by physical touch, and movement through the channel was recorded for 2 min. The measurements were repeated 3 times per replicate.

### Part 2: follow-up experiment

#### Test organism and test compound

Based on the results of the Hackathon, a follow-up experiment was conducted using *C. riparius. C. riparius* was cultured in a climate room (21 ± 1 °C; 16:8 light to dark cycle) following preferred settings as proposed by OECD 219 (OECD 2004). Eggs were collected for hatching 6 days before the experiment and stored in an aquarium filled with M7 medium and inorganic sand (grain size of 0.01–2 mm; Décor Son, Praxis, Leiden, the Netherlands). The eggs were stored at 20 ± 1 °C and natural light. Sulfoxaflor (97.8%; CAS No. 946578-00-3) was purchased from AccuStandard (New Haven, CT, USA), and 5 different treatments of sulfoxaflor and a control were used. The nominal concentrations were 6.25, 12.5, 30, 80, and 160 μg/L.

#### Exposure setup

For the experiment, 360 newly hatched larvae (< 48 h) were collected at Day 0 and divided at random with 5 larvae in each test beaker (*n* = 12 beakers/treatment). Sample size was determined *a priori* to ensure adequate numbers of replicates and ad hoc, using the software G*Power 3.1.9.4 (*α* = 0.05, *β* = 0.20, CES = 25%; Franz Faul, Kiel, Germany). A total of 12 replicates with 5 larvae per test vessel were chosen to have maximal statistical power using limited amount of organisms and within the physical constraints of the experiment. Each beaker was filled with 85 grams of sand and topped with M7 medium spiked with sulfoxaflor (ratio: 4:1 between water and sand; OECD 2004), corresponding to a surface area of 3.9 cm^2^/organism. A trouvit/tetraphyl mix in a 20:1 ratio was added to each test beaker as food at Day 0, corresponding to 0.5 mg/organism/day (Postma et al. 1995). On Day 7, 50 mL of exposure medium was renewed with freshly made sulfoxaflor concentrations to maintain water conditions and sulfoxaflor concentrations throughout the test duration. pH, dissolved oxygen, and conductivity were all measured at start and end of the experiment. The test used a climate chamber (Memmert Peltier-cooled incubator IPP110plus) at 21 °C and a 16–8 light/dark cycle. All beakers were connected to an aquarium pump and aerated lightly throughout the exposure. The exposure was run for 21 d or until all larvae in one replicate had emerged. Energy-related and biomechanistic endpoints were measured on Days 0, 3, and 9.

#### Determination of energy-related endpoints

On Day 0 of the experiment, 60 larvae were randomly selected to determine the average length at the start of the experiment. After 3 days of exposure, 8 replicates/treatment were used to measure activity and growth. Measured larvae were returned to test beakers afterwards for the remainder of the experiment. On Day 9, mortality, growth and changes in locomotive behaviour were measured in all replicates. For the mortality assessment, the content of each beaker was emptied into a larger tray for inspection. The larvae were considered alive if they responded to physical stimuli at the time of sampling or were not visibly degrading. For all 3 sampling times, growth was determined for the retrieved larvae by measuring their length from head to tail in ImageJ 1.53e (Wayne Rasband, Maryland, USA; Abramoff et al. 2004; Richardi et al. 2015).

After Day 9, the content of each beaker was transferred back into each beaker, including respective larvae. It was left in the climate chamber to observe any potential changes in the emergence of the adults. Continuation of replicates was chosen over discarding to make the most use of resources. Based on strict observations before the experiments as well as during the experiment, it was found that the handling of the larvae did not disturb emergence. Based on the high water solubility of sulfoxaflor, disrupting the sediment was not considered a threat to the chemical steady-state equilibrium. Yet, the overlying water was exchanged throughout the test period, as well a chemical analyses were performed to observe any potential changes. Each beaker was covered with parafilm to trap the adults. The beakers were checked daily for 12 days for emerging adults, which were subsequently removed. Furthermore, on Day 21, the total number of emerged adults was validated by counting the number of empty pupa.

#### Determination of biomechanistic endpoints

During the Hackathon, it was observed that sediment disturbance leads to increased activity of the larvae, meaning that buried larvae would move to the sediment surface and hence be visible. On Day 3, activity was assessed using this technique (*n* = 8). Larvae were deemed active if they could be retrieved from the sediment using visual inspection. Retrieval of the larvae was done by pipetting and gently stirring the sediment until all active larvae were retrieved upon visual inspection of the sediment surface. Stirring continued 2 min after the last larva was found to ensure the larvae had the proper time to react to the stimuli from stirring the sediment. Furthermore, the animals were considered active if they were moving when touched.

After mortality and growth assessment on Day 9, alive larvae were transferred into 6-well plates (*d* = 3.54 cm), with one larva per well containing 10 mL of exposure medium and no sediment. The larvae were left to acclimatise for at least 10 min, whereafter, swimming behaviour was recorded for 10 minutes (Panasonic HDC-SD90 Full HD camera, Panasonic, Osaka, Japan). Larvae were tracked using a tracking program (Ethovision XT, version 14; Noldus, Wageningen, the Netherlands) by calculating the movement of each individual based on the difference between the frames (frame per second = 5). Measured endpoints were the average velocity (*v*), number of seconds spent in fast-moving pace (> 25 mm s^-1^), and slow/no-moving pace (< 5 mm s^-1^). The limit for swimming behaviour at a velocity of 25mm/s was selected to include all swimming events as described by Brackenbury (2000). By utilising automatic software tracking, we ensured to get any variation in movements down to 0.2 s. Importantly, the outcomes of the tracking program were checked against 5 control replicates by matching the frames in which high velocities were detected by the program with visual inspection of the corresponding videos. Some mistracing was observed during the analysis due to larvae reflection interfering with the larvae recognition of Ethovision. This was solved by selecting an upper limit of movement between each frame of 25 mm, ensuring no mistracking.

### Chemical analysis

Chemical analysis for sulfoxaflor was done in the diluted stock solutions before they were transferred into the experimentation setups. The diluted solutions were left at room temperature throughout the experiment and were treated similarly to the test beakers. Samples were taken on Days 1, 3, 4, and 7. All solutions were made from a stock solution of sulfoxaflor stored at < 5 °C, as sulfoxaflor is considered stable in water (US EPA 2019). For the chemical analyses in Part 2, 2 extra replicates/treatments were included without any animals, and sampling of the overlying water took place in these replicates. The sampling took place on days 0, 1, 3, 5, 9, and 15 to observe if changes in concentrations of sulfoxaflor throughout the experiment occurred. Samples were stored in a freezer (− 20 °C) until chemical analysis could take place. Sulfoxaflor has a high water solubility of 568 mg/L at 20 °C (EFSA 2014), and with a coefficient of adsorption of 7–74 mL/g (US EPA 2019), we do not expect sulfoxaflor to partition into the sediment. Furthermore, the formation of U-shaped borrows in chironomid species results in the majority of uptake of contaminants from the water phase (Hare et al. 2001). Considering the above, the water phase was expected to be the main route of exposure for the organisms.

Chemical analysis of sulfoxaflor was conducted using an LC system (Waters Aquity I-class) coupled to a mass spectrometer (ScieX Qtrao 6500) equipped with a corresponding column (Waters ACQUITY UPLC BEH C18; 2.1 mm × 50 mm, particle size: 1.7 μm). The samples were separated using two eluents: eluent A (95% H_2_O, 5% ACN, 0,1% formic acid) and eluent B (95% ACN, 5% H_2_O, 0,1% formic acid) in a gradient for a total of 6 minutes (flow: 0.60 mL min^-1^; see *supplementary information*). The output was scanned using multi-reaction monitoring in positive ion mode (MRM). The same sulfoxaflor stock solution from the exposure scenarios was used as the standard, from which a calibration with a range of 0–200 μg/L sulfoxaflor was made. All samples were 1 mL and done in duplicates; averages were used to calculate actual sulfoxaflor concentrations. To reduce uncertainties, an internal standard of sulfoxaflor-d3 (Toronto Research Chemicals Inc.) was added to all the sample vials in a concentration of 10 μg/L. Lastly, 3 instrumental replicates were performed of all the samples (further details can be found in *supplementary information*).

### Statistical analysis

Statistical analyses for part 1 were performed by each student group. Normality was checked using visual inspection of QQ plots or a Shapiro-Wilk test depending on sample size; a Levene’s test followed both methods to assure homogeneity in variance across the different treatments. For normally distributed data, including growth and locomotion, one-way ANOVA tests were applied to determine statistical significance, followed by Dunnett’s post hoc test to compare treatments to controls. In non-normally distributed data, data were log-transformed before being analysed using a mixed linear model (Poisson distribution). Additionally, Kruskal-Wallis tests were used to determine significance of the data where a log transformation did not satisfy normality. All data are given as mean change from control ± standard error (SE). G*Power 3.1.9.4 (Franz Faul, Kiel, Germany) was used post hoc for power analysis (α = 0.05, *n* = 7, CES = 25%). Furthermore, for data showing signs of a dose-dependent relationship, dose-response curves were performed using R Software (RStudio 4.1.3; Posit, Boston, USA) with log-log interpretations in the drc-package (version 4.1.3).

For part 2, survival and total emergence were found per replicates (*n* = 12), whereas behavioural data was analysed per individual (*n* = 60) to adequately consider the individual variations between each larvae. Data from each endpoint and concentration were tested individually for normality by visually inspecting QQ plots; all endpoints showed normal distribution. Homogeneity of variance was attested using Levene’s test. Statistical significance was tested using one-way ANOVA tests with an *α* = 0.05 using GraphPad Prism 9.0.0 (GraphPad Software, Inc., La Jolla, USA) followed by Dunnett’s multiple comparison tests to determine significant differences between each treatment and control. Dose-response curves were made in R 2022.07.0 (Posit, Boston, USA ) using the tool dmr found in the drc-package. The curves were based on responses found as decrease or increase in percentages from control and actual concentrations. Dose-response models were fitted using a two-parameter log-logistic function for survival, total emergence, emergence per larvae and activity; for the rest, a four-parameter log-logistic function was found to be the most suitable.

## Results

### Water chemistry

Water chemistry parameters in the exposure solutions were not impacted by the addition of sulfoxaflor in either part 1 or part 2. For all tests performed in the hackathon, the pH was between 7 and 8 at the start of the experiment. Dissolved oxygen was initially reduced by the addition of DECOTABs in 30 out of 50 test beakers with *A. aquaticus*. This was corrected on Day 2, after which dissolved oxygen was between 60 and 100% throughout the test period. Concentrations of sulfoxaflor showed to be stable over time, with mean recoveries of 70–109% for the first 4 days. However, for nominal concentrations of 25 μg/L and 50 μg/L, recoveries of sulfoxaflor were down to 56 and 26%, respectively, after renewal of the medium, indicating new solutions might have been suboptimal (see *supplementary information* for all details). For consistency, we kept the results for part 1 as nominal concentrations.

For part 2, measured pH values ranged from 6.7 to 7.2, and conductivity ranged from 667 to 697 μS/cm at start of the experiment. Dissolved oxygen was above 90% saturation. By Day 7, pH was between 7.7 and 8.7, and conductivity remained constant throughout the duration, with the exception of the control at 993 μS/cm. The mean recovery of sulfoxaflor was between 31 and 42 %, except for the lowest concentration, where a mean recovery of only 14% was determined. Low recoveries could be due to degradation through photolysis (Yang et al. 2022); however, only limited change in measured concentrations of sulfoxaflor over time was observed (mean concentrations over time are provided in *supplementary information*). This indicates that transformation of sulfoxaflor over time is likely not the leading cause of low recoveries. Furthermore, only limited partitioning to the sediment was expected, which is in agreement with the comparably low recoveries observed for the samples taken before adding the dilutions to the test vessels. To accommodate the discrepancy between intended and actual concentrations, all results for part 2 are based on actual concentrations: 0.90 (± 0.07), 3.90 (± 0.18), 11.1 (± 0.17), 33.9 (± 0.25), and 67.2 (± 1.15) μg sulfoxaflor (SE)/L. No sulfoxaflor was measured in the controls.

### Part 1: outcome of the hackathon

**Fig. 1:**
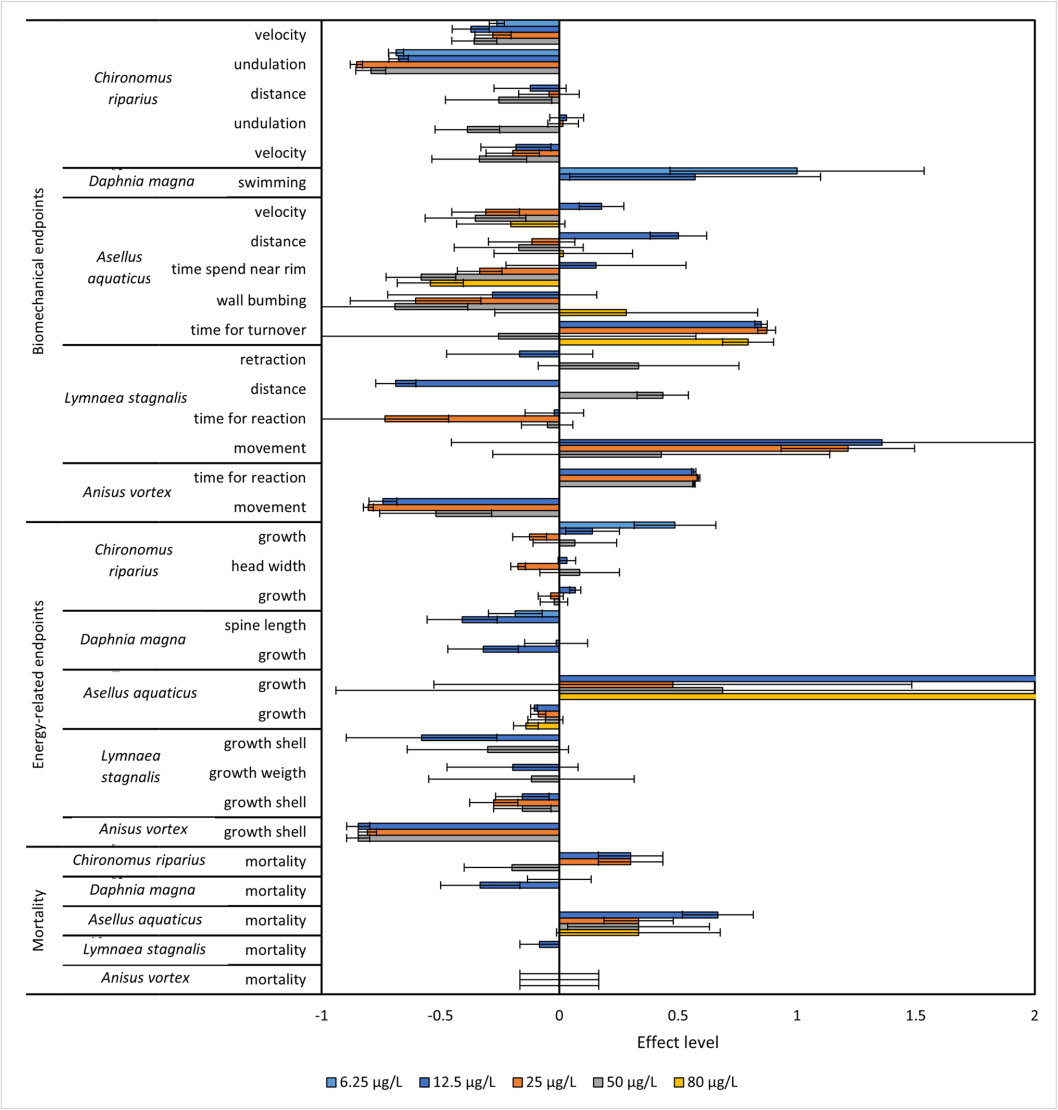
Normalised values compared to controls from part 1. Endpoint/organism combination is shown on the *y*-axis. The *x*-axis shows the effect level normalised to the mean of controls. Positive values denote a positive change for the endpoint compared to the control. Negative values depict negative effects and a decrease in the endpoint compared to controls. Error bars show standard error. Colours indicate the nominal concentrations of sulfoxaflor in which the organisms were exposed to

High variances were observed in all endpoints for all 5 species (Fig. 1). Furthermore, no significant monotonic positive or negative relationship was observed. As a result, only general trends on potential impacts could be made. Importantly, this study focused on sub-lethal endpoints and only limited mortality was observed for all 5 species (Fig. 1*)*.

For the standard endpoints, tendencies headed in different directions, with large variances and inconsistent results across the groups. Declines were observed in some tests (although not statistically significant); however, no dose-dependent effects for survival and growth were found for any species (Fig. 1).

Likewise, biomechanistic endpoints showed, for the most part, contrasting effects in both positive and negative directions and high variances. One exception, biomechanistic endpoints for *C. riparius*, including undulation and velocity, indicated potential adverse effects, as all assessed behavioural endpoints showed degrees of adverse effects (Fig. 1). Both student groups found that mean velocities of movements were impacted at 6.25 μg/L, and undulation was impacted negatively at 6.25 μg/L for one group; the other group only saw adverse effects at 50 μg/L (Fig. 1). Based on the results of the Hackathon, biomechanistic endpoints tested on *C. riparius* were found to be the most sensitive and consistent. Hence, we decided to include *C. riparius* in a follow-up experiment to improve the measurement method of locomotion.

### Part 2: follow-up experiment

**Fig. 2:**
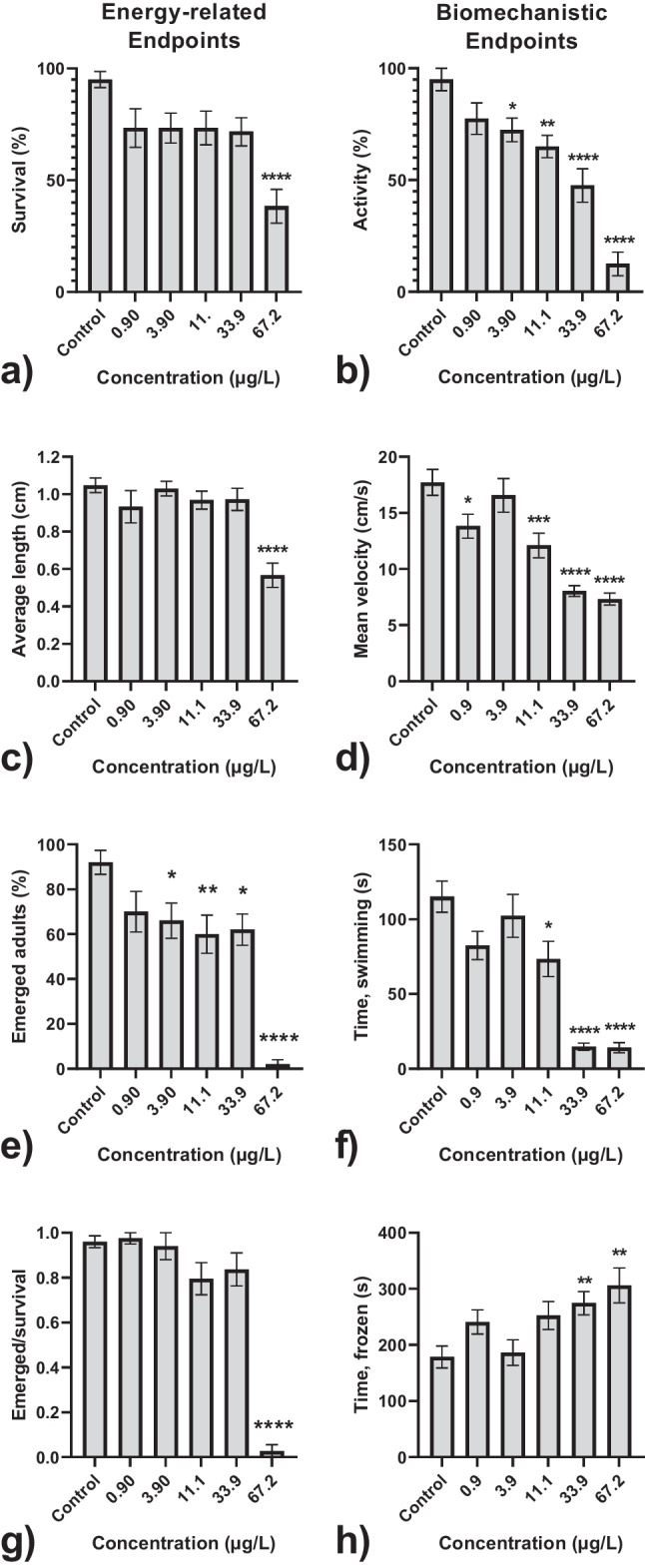
Effects of sulfoxaflor on selected endpoints (+/− SE) on *C. riparius* larvae. **a** Mean survival on Day 9 (*n* = 12). **b** Mean activity measured as fleeing response in sediment on Day 3 (*n* = 8). **c** Mean length on Day 9 (*n* = 10). **d** Mean velocity in water phase on Day 9 (*n* = 12). **e** Mean emergence of adults (*n* = 10). **f** Mean duration of time spent swimming on Day 9 (*n* = 12). **g** Emergence of adults per surviving larvae on Day 9 (*n* = 10). **h** Mean duration of time spent not moving on Day 9 (*n* = 12). Significance level is determined with a one-way ANOVA (difference from control *P ≤ 0.05, **P ≤ 0.01, and ***P ≤ 0.001)

Adverse effects were found for all 8 endpoints at the highest concentration of sulfoxaflor (67.2 μg/L; Fig. 2). Significant reductions were only found for mortality and growth for the highest concentration (Fig. 2), whereas total emergence on Day 21 showed effects at concentrations as low as 3.90 μg/L. However, emergence per surviving larvae on Day 9 showed no effects at concentrations lower than 67.2 μg/L, indicating that effects on total emergence could, to a degree, be caused by mortality in the larval stage. In contrast, the biomechanistic endpoints seemed to show more consistent effects at lower concentrations (Fig. 2). Mean velocities of the larvae and the time the larvae spent swimming were found to be the most sensitive, with significantly reduced mobility of the larvae already at 11.1 μg/L.

This difference in sensitivity across different endpoints was further highlighted by the dose-response relationships and corresponding EC_50_ values (Table 3). These results indicate that overall biomechanical endpoints are a more sensitive indicator of effects compared to energy-related endpoints. Based on the EC_50_ values, the order of sensitivity was mean velocity > fast pace > activity > no mobility. Only total emergence was within the same sensitivity range of energy-related endpoints compared to the biomechanistic endpoints. Growth and survival were found to have low sensitivities for effects, with an EC_50_/LC_50_ value of 105 μg/L and 116 μg/L, respectively.

**Table 3:** All LC_50_/EC_50_ values in μg sulfoxaflor /L (95 % confidence interval) based on dose-response curves. The responses are found as decreases in fitness relative to control means. Dose-response models are fitted using a two-parameter log-logistic function when suitable and a four-parameter log-logistic function for the rest

Endpoint type	Endpoint (day of measurement)	EC_50_ in μg /L (95 % confidence interval)
Mortality	Survival (day 9)	116 (0–304)
Energy-related endpoints	Growth (day 9)	105 (0–820)
	Total emergence (day 21)	18.3 (7.62–29.0)
	Emergence/survival	42.6 (31.1–54.1)
Biomechanistic endpoints	Activity (day 3)	20.1 (12.2–28.0)
	Mean velocity (day 9)	10.6 (0.40–20.8)
	Fast pace (day 9)	11.4 (0–54.8)
	No mobility (day 9)	28.5 (0–62.6)

**Fig. 3:**
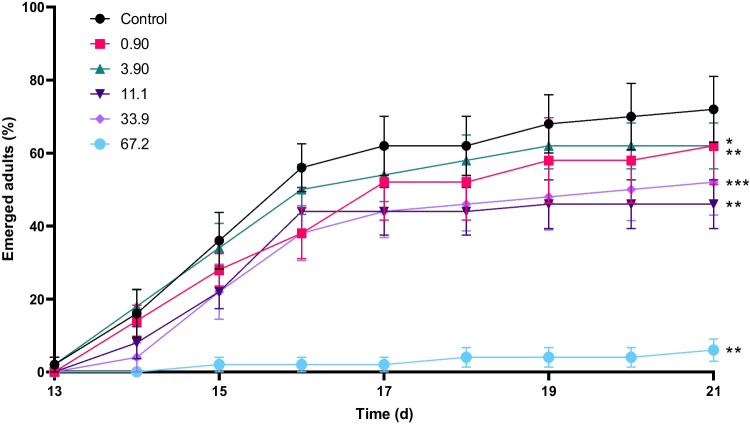
Emergence of adults in percentage of added larvae in each beaker over time (*n* = 12). Colours indicate actual concentrations described in μg/L sulfoxaflor. The error bars show standard error. Significance is determined against controls by one-way ANOVA test followed by a Dunnett’s multiple comparison test (*P ≤ 0.05, **P ≤ 0.01, and ***P ≤ 0.001)

Moreover, we observed a delay in emergence as well as a decrease in total emergence when larvae were exposed to all concentrations of sulfoxaflor compared to controls (Fig. 3). The daily number of emerging adults is the preferred endpoint included in the OECD Test Guideline 219 (OECD 2004). Based on this standard endpoint, sulfoxaflor had apparent effects on *C. riparius*, starting at 0.90 μg/L. At 67.2 μg/L, less than 5% of initial larvae were able to emerge as adults within 21 days. However, when expressing total emergence per surviving larvae, only the highest concentrations induce significant changes (Fig. 2).

## Discussion

### The hackathon

Here, we present the results of a Hackathon in environmental toxicology (to our knowledge, the first of its kind). The aim was to determine sensitive endpoints of several freshwater organisms to exposure to a neurotoxic insecticide. The hackathon was done with two main purposes in mind: to gain valuable data on the ecotoxicity of a novel neurotoxic insecticide, sulfoxaflor, and as part of a master’s education program, to train students in the field of ecotoxicity.

#### Toxicity testing

Previous studies have found that the toxicity of neurotoxic insecticides determined by standard testing can be underestimated (Rubach et al. 2011; Raby et al. 2018; Barmentlo et al. 2019; Liu et al. 2021). Concentrations tested in this hackathon suggested changes in several energy-related and biomechanistic endpoints, which are important for population fitness, even though no increase in mortality was observed for any species. Notably, high variances were observed in all endpoints (Fig. 1), likely due to the low number of replicates, the limited lab experience of the students, and interpersonal differences in measurements and interpretation. These increased variances are a trade-off from the high quantity of data generated in the hackathon. For *C. riparius*, our data suggest that swimming behaviour of the larvae is altered by exposure to sublethal levels of sulfoxaflor. This can greatly impact the larvae's overall fitness (Pestana et al. 2009; Langer-Jaesrich et al. 2010). *C. riparius* was hence found a promising candidate for further investigation. Furthermore, an EC_50_ value for *C. riparius* for growth was 10.7 μg/L after 9 days of exposure. This is in agreement with previous literature using chironomids sp., suggesting that the overall exposure setup yielded realistic results. Liu et al. (2021) found effects on the emergence and growth of *Chironomus kiinensis* at concentrations as low as 20 μg/L. The fact that sensitivities of chironomids sp. found in the hackathon are in similar range to other studies using similar neurotoxic chemicals suggests that even with the high variation we observed, a Hackathon event using students is suitable for pilot studies.

##### Hackathons in teaching

The aim of the master course, in which the Hackathon took place, was to teach the students the fundamentals of ecotoxicity and to be able to assess risk of environmental contaminants. To achieve this, we encouraged the students to actively participate with their own ideas; compared to traditional lectures, actively engaging students has been shown to increase learning efficiency (Deslauriers et al. 2019). Over the weeks, we observed increased student responsibility, resulting in increased productivity and quality of the experimental solutions.

We believe this teaching method can spark interest in ecotoxicology in the coming generation of researchers, making newcomers join the pursuit of knowledge in environmental sciences. Two comments we received in the course evaluation after finalising the course were: “[The] course triggers further study and interest for students” and “… [the] practical part of the course is really nice because you really learn what it is like to be a scientist in action” (see complete course evaluation in *supplementary information*). Both comments highlight that this kind of teaching can generate inspiration in the students, which more traditional teaching methods might lack (Hawtrey 2007; DeHaan 2011).

**Fig. 4:**
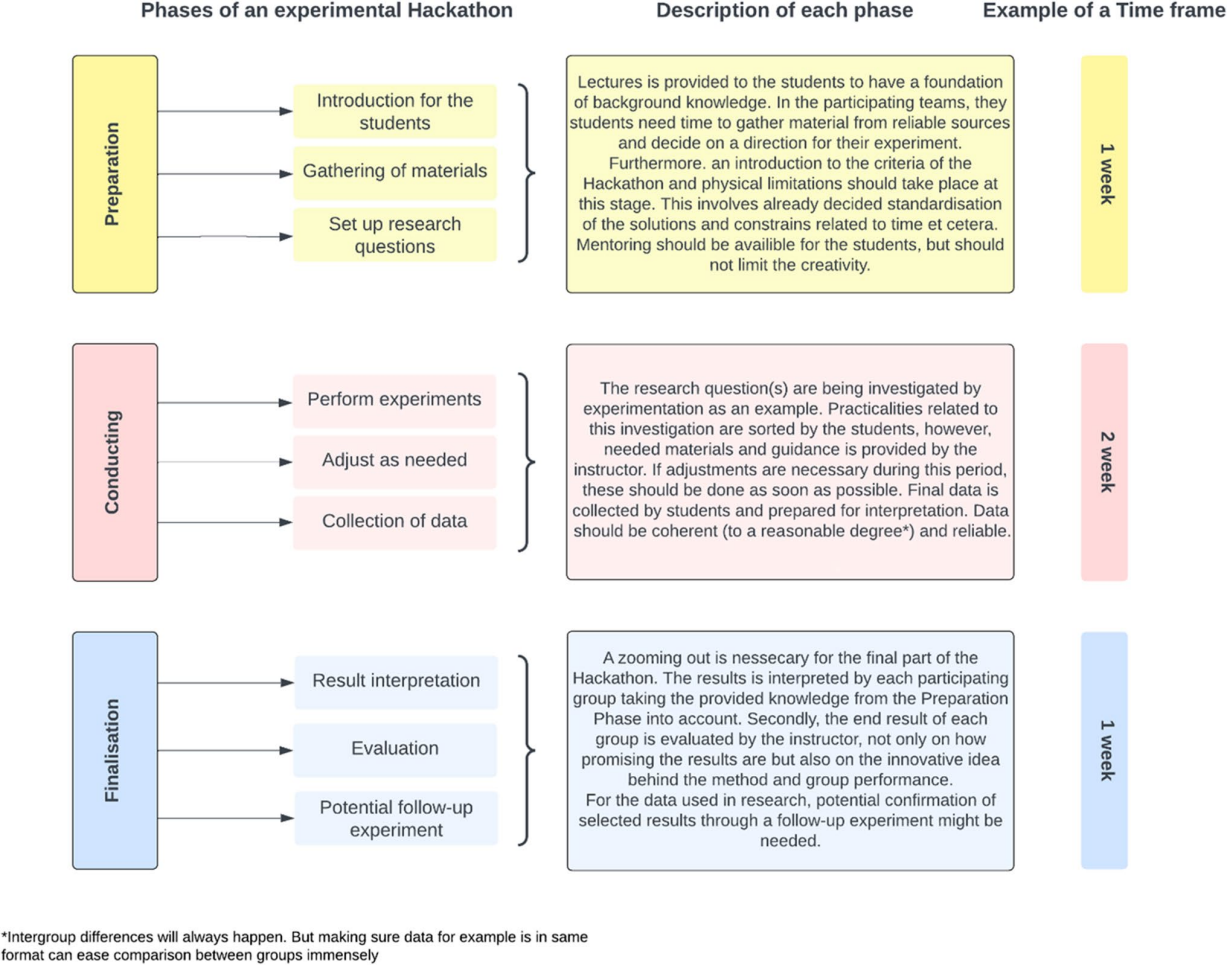
Proposed framework for using Hackathons in teaching environmental sciences

In Fig. 4, we propose a framework to be implemented when considering hackathons for ecotoxicity studies.

The framework is proposed based on our experiences with a 4-week master course (Fig. 4 and Table 1). However, the concepts can be translated into numerous other topics, levels of education, and durations of time. The framework describes 3 phases of an experimental hackathon: preparation, conducting, and finalisation. The preparation phase lays the foundation of the Hackathon and is essential to introduce the students to the overall aim of the hackathon. Already in the beginning, it can be important to have some guidelines on standardisations between different groups, such as similar concentrations of the same chemical and criteria for minimum inclusions of endpoints. Furthermore, the inclusion of mortality was used for quality assurance and could be compared to standard protocols (OECD 2004; OECD 2012). Importantly, standardisation should not limit creativity and should require individual assessments based on the overall goal of the Hackathon (Cropley 2015; Yarmohammadian et al. 2021).

For the conducting phase, the instructors can take a step back and leave the execution up to each group. This provides a healthier power balance between participants and researchers, increasing the success rate of an impactful outcome (Roura 2020). Guidance should be readily available throughout the experimental period. We experienced frequent evaluations of students and instructors during the Hackathon, which improved the overall results and drove growth and development (Medina et al. 2019; see *supplementary information*). Lastly, the finalisation phase includes results interpretation, putting the individual results obtained by each group into perspective and reflection. We suggest that each group prepare a presentation on selected findings. This gives the students valuable experience in scientific presentation and the challenge of selecting important and ecologically relevant findings.

### Follow-up experiment using *C. riparius*

The results of the hackathon showed that biomechanistic endpoints were more sensitive compared to most of the energy-related endpoints or mortality. This is in line with findings that neurotoxic chemicals are suspected of inducing changes in biomechanical behaviour, including mobility (Augusiak and Van den Brink 2016), predator escape response (Pestana et al. 2009), and feeding activities (Langer-Jaesrich et al. 2010).

Although the hackathon provided evidence that *C. riparius* is a sensitive species, there were considerable issues with statistical power due to high variance and relatively low sample sizes. To create a more robust experimental design, the number of replicates and tracking time of the biomechanistic endpoints was increased (e.g., tracking duration *t* = 10 min/replicate). The estimated EC_50_ values were consistent with the student data. Mean velocity showed the highest sensitivity, closely followed by the time spent either moving or frozen. These are ecologically relevant endpoints, as *C. riparius* larvae depend on a fleeing response when a predator is nearby (Hölker and Stief 2005). Therefore, changes in mobility can lead to severe adverse impacts on the population level by increased mortality from predation (Weis et al. 2001; Langer-Jaesrich et al. 2010). This highlights that biomechanistic endpoints of *C. riparius* are not only sensitive and practical but also environmentally relevant (Pestana et al. 2009).

A crucial other finding is that emergence over time was more sensitive, with a significantly lowest observed effect concentration (LOEC) of 0.90 μg/L, compared to total emergence (LOEC of 3.90 μg/L ). Delays in emergence can have disruptive consequences on overall population levels of insects (Dewey 1986), especially as chironomids have a short lifecycle, including the adult stage (Karima 2021). During the aerial stage, mating occurs in large swarms, where the males attract the females. The larger the swarm, the more attractive to the females, allowing high mating rates (Karima 2021). Changes in time of emergence, even a matter of days, can, therefore, directly impact the reproduction rate of chiromidae populations, making this a vital endpoint for toxicity studies (Barmentlo et al. 2021). We found that the emergence of alive larvae was significantly impaired in the highest concentration (67.2 μg/L), with only a minimal number of adults in this concentration. The EC_50_ was in this study found to be 42.6 (31.1–54.1) μg/L for emergence after 21 days of exposure to sulfoxaflor. Liu et al. (2021) reported that emergence was significantly reduced at concentrations of 20–79 μg/L, and 90 μg/L resulted in significantly delayed emergence (Liu et al. 2021). This confirms that our effect values are comparable to previously reported values.

Emergence is a standard endpoint described for chironomids in OECD Test Guideline 219 (OECD 2004). However, the endpoint takes a minimum of 13 days to reach. The biomechanistic endpoints on swimming behaviour, velocity, and undulation were shown to be sensitive in a shorter experimental duration. Importantly, behavioural endpoints are ecologically relevant. For example, Langer-Jaesrich et al. (2010) showed increased predation by *Danio rerio* when *C. riparius* larvae were exposed to sublethal concentrations of chlorpyrifos because of altered burrowing behaviour. Hence, changes in locomotion can be lethal for chironomid larvae in the environment, where predators are present (Langer-Jaesrich et al. 2010).

Overall, our results suggest that standard testing does not adequately reflect the potential effects of sulfoxaflor on *C. riparius*. Measuring biomechanistic alterations is a promising addition in toxicity testing, as they can be measured relatively easily and are ecologically relevant (Sánchez-Bayo et al. 2016; Brooks et al. 2021).

## Conclusions

In conclusion, we demonstrate here that participatory research executed in a hackathon provides key data that could be used to develop sensitive endpoints. This approach is especially valuable for those cases where standard toxicity testing might not be sufficient to address potential environmental effects. Importantly, following a hackathon, more refined experiments can be used for the most promising endpoints. For that purpose, a follow-up experiment was done to determine accurate and robust dose-response curves for sulfoxaflor to a sensitive aquatic organism using biomechanistic endpoints. We demonstrated that changes in locomotion of *C. riparius* larvae measured as their mean velocity in water showed impacts at levels 5–10 times lower compared to mortality.

This highlights the benefit of using a hackathon in several ways. Firstly, from a scientific screening perspective, hackathons can efficiently obtain a large amount of data. Secondly, from the student’s perspective, it had clear added value as they felt engaged in working with a real and relevant challenge. Suppose this framework is implemented more widely in science education. In that case, we predict that we can successfully inspire a whole new generation of stakeholders in society, whether in science, governance, or the industry, to engage with the field of environmental toxicology.

### Supplementary information

ESM 1 (DOCX 17 kb)

ESM 2 (PDF 694 kb)

ESM 3 (DOCX 284 kb)

ESM 4 (XLSX 82 kb)

ESM 5 (XLSX 171 kb)

ESM 6 (DOCX 21 kb)

## Data Availability

Supplementary information is provided. The raw data files can be obtained upon request.
