# Supplementary material for: Participatory hackathon to determine ecological relevant endpoints for a neurotoxin to aquatic and benthic invertebrates
Source: Environ Sci Pollut Res Int. 2024 Feb 28;31(15):22885–99. doi: 10.1007/s11356-024-32566-w (PMC10997722; doi:10.1007/s11356-024-32566-w)
Supplement: Supplementary file 1 — (DOCX 17 kb) [file 11356_2024_32566_MOESM1_ESM.docx]

**Participatory Hackathon to determine ecological relevant endpoints for a neurotoxin to aquatic and benthic invertebrates**

Sofie B. Rasmussen^1, *^, Thijs Bosker ^1,2^, Giovani G. Ramanand^1^, Martina G. Vijver^1^

^1^ Institute of Environmental Sciences, Leiden University, P.O. Box 9518, 2300 RA Leiden, the Netherlands

^2^ Leiden University College, Leiden University, P.O. Box 13228, 2501 EE, The Hague, the Netherlands

^*^Corresponding author, Institute of Environmental Sciences, Leiden University, P.O. Box 9518, 2300 RA Leiden, the Netherlands. Email: [a.s.b.rasmussen@cml.leidenuniv.nl](mailto:a.s.b.rasmussen@cml.leidenuniv.nl), tel.: +45 20334344

**For submission in Environmental Science and Pollution Research**

DECOTAB protocol – Martin van der Plas & Tom Nederstigt – 9-12-2021

**Equipment**

- Scissors
- Electric shredder
- 500 µm and 250 µm sieve
- Analytical balance
- Heating plate with magnetic stirrer
- 1 L Schott bottle
- Heat resistant glove
- Scalpel and cutting mat or punch/perforator (optional if you want to cut them to size after preparing)

**Materials for ~ 1 L of decotab mixture**

- 60 g of plant material (e.g. organic hay)
- 20 g agar
- 1 L MiliQ or demineralized water

1. Use scissors and electric shredder to cut plant material as fine as possible. This is tedious and can take a long time, and it is best to do many batches and accept that only a small part of the used material will end up within the preferred size range. Leftovers from step 2 may be mixed and cut again. Make sure the electric shredder does not overheat
2. Deposit shredder plant material on the stacked 500 µm and 250 µm sieve. Manually sieve by tapping and shaking, but avoid pushing the material into the sieve (this will only cause clogging)
3. Repeat step 1 and 2 until 60 g of plant material < 250 µm is obtained
4. Heat 1 L of MiliQ or demineralized water on the heating plate to ~ 95 ºC
5. Add the magnetic stirrer and make sure it rotates at maximum speed without moving out of center
6. Add 20 g of agar
7. Very slowly add 60 g of the < 250 µm plant material. Note that this is where you add all that plant material that you worked for, so do it slowly and carefully. Reheating of the solution is not an option if things go wrong. It is crucial to maintain proper rotation of the magnetic stirrer. If this proves difficult, use a heat resistant glove and take the bottle of the heat source in intervals and shake. To do so, put the cap on the bottle briefly, but **keep in mind that pressure builds up quickly**. Therefore, burp the bottle carefully every few seconds. Note that a homogeneous suspension is what you are after.
8. Pour the mixture into the mold whilst still hot and leave to solidify.
9. Cut decotabs to size and store in the fridge until ready for use. Note that storing in the freezer is not ideal as they may fall apart. However, storing in the fridge will result in molt within a few weeks, so don’t store for too long.
10. Prior to use (and afterwards if desired), dry a subset of DECOTABs (ideally more than 10) in a stove over the course of 2 days at 70 ^o^C in pre-weighed weighing boats to determine dry weight.
