# Supplementary material for: Participatory hackathon to determine ecological relevant endpoints for a neurotoxin to aquatic and benthic invertebrates
Source: Environ Sci Pollut Res Int. 2024 Feb 28;31(15):22885–99. doi: 10.1007/s11356-024-32566-w (PMC10997722; doi:10.1007/s11356-024-32566-w)
Supplement: Supplementary file 2 — (PDF 694 kb) [file 11356_2024_32566_MOESM2_ESM.pdf]

Participatory Hackathon to determine ecological relevant endpoints for a neurotoxin to aquatic and benthic invertebrates

Sofie B. Rasmussen[1, *], Thijs Bosker [1,2], Giovani G. Ramanand[1], Martina G. Vijver[1]

[1] Institute of Environmental Sciences, Leiden University, P.O. Box 9518, 2300 RA Leiden, the Netherlands
[2] Leiden University College, Leiden University, P.O. Box 13228, 2501 EE, The Hague, the Netherlands

[*]Corresponding author, Institute of Environmental Sciences, Leiden University, P.O. Box 9518, 2300 RA Leiden, the Netherlands. Email: a.s.b.rasmussen@cml.leidenuniv.nl, tel.: +45 20334344

For submission in Environmental Science and Pollution Research

Ecotoxicology - 4313ETX19

Ecotoxicology ()
No. of responses = 33

Survey Results

Legend

Question text

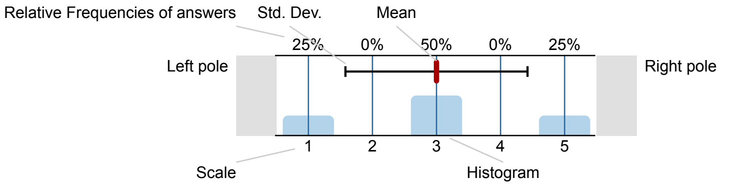

n=No. of responses
av.=Mean
dev.=Std. Dev.
ab.=Abstention

General -

You took this course as part of the programme:

n=33

Artificial Intelligence	0%
Astronomy	0%
Bioinformatics (I&B)	0%
Biology	93.9%
Bio-Pharmaceutical Sciences	3%
Business Studies	0%
Chemistry	0%
Computer Science	0%
Computer Science & Economics (I&E)	0%
Farmacie	0%
Governance of Sustainability	3%
ICT in Business	0%
Industrial Ecology	0%
Life Science and Technology	0%
Mathematics	0%
Media Technology	0%
Molecular Science and Technology	0%
Physics	0%
Statistical Science	0%
Statistics and Datascience	0%
other	0%

I am registered as a:

n=33

full time student	100%
part time student	0%
guest/contract student	0%

I attended this course as a:

bachelor's student	0%	n=33
double-bachelor's student	0%	
pre-master's student	0%	
master's student	100%	

In my degree programme this course is a:

compulsory course	60.6%	n=33
elective / minor's course	39.4%	
honours' course	0%	
other	0%	

Course specific

My **overall rating** of this course on a scale from 1 to 10 (Dutch grading scale) is:

1 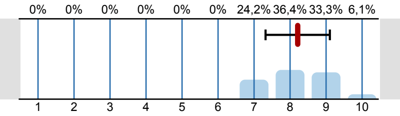 10

n=33
av.=8,2
dev.=0,9

In terms of difficulty, the course is:

too easy 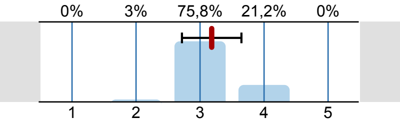 too difficult

n=33
av.=3,2
dev.=0,5

I estimate the percentage of class sessions which I attended well-prepared to be approximately:

less than 25%	3%	n=33
26-50%	6.1%	
51-75%	15.2%	
76-99%	63.6%	
100%	12.1%	

Compared to the prescribed study load (1 EC = 28 hours including contact hours), the actual study load of this course is:

too light 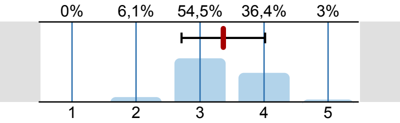 too heavy

n=33
av.=3,4
dev.=0,7

The lecturer(s)/instructor(s) in this course teach(es) well.

totally disagree 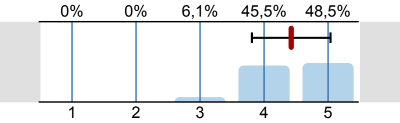 totally agree

n=33
av.=4,4
dev.=0,6

The instructional methods (lecture, seminar, practical/lab work, etc.) are well matched to what you should have mastered at the end of the course.

totally disagree 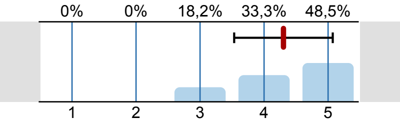 totally agree

n=33
av.=4,3
dev.=0,8

The provision of information for this course was good.

totally disagree 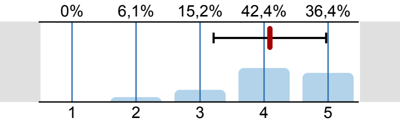 totally agree

n=33
av.=4,1
dev.=0,9

I could easily get an answer to my questions outside of normal contact hours.

totally disagree 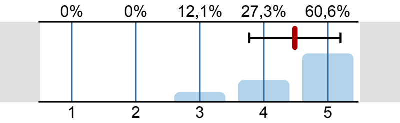 totally agree

n=33
av.=4,5
dev.=0,7

Question	Left pole	1	2	3	4	5	Right pole	Statistics
The course had the right structure/organisation to enable me to master its contents.	totally disagree	0%	6,1%	18,2%	42,4%	33,3%	totally agree	n=33 av.=4 dev.=0,9
This course stimulates me to pursue my studies.	totally disagree	0%	0%	18,2%	27,3%	54,5%	totally agree	n=33 av.=4,4 dev.=0,8
The online parts of this course provide effective support for my learning process.	totally disagree	3,1%	3,1%	15,6%	43,8%	34,4%	totally agree	n=32 av.=4 dev.=1 ab.=1

Study material

Question	Left pole	1	2	3	4	5	Right pole	Statistics
The course materials (literature, video, podcast, Brightspace, etc.) are well matched to what you should have mastered at the end of the course.	totally disagree	0%	0%	24,2%	39,4%	36,4%	totally agree	n=33 av.=4,1 dev.=0,8
The quality of the study material was good.	totally disagree	0%	0%	21,2%	54,5%	24,2%	totally agree	n=33 av.=4 dev.=0,7
The study material on Brightspace or other digital learning environment was easy to access.	totally disagree	0%	6,1%	3%	45,5%	45,5%	totally agree	n=33 av.=4,3 dev.=0,8
There was sufficient practice material available.	totally disagree	0%	24,2%	30,3%	21,2%	24,2%	totally agree	n=33 av.=3,5 dev.=1,1

Testing

Question	Left pole	1	2	3	4	5	Right pole	Statistics
I knew in good time what I had to master for a test.	totally disagree	0%	15,2%	27,3%	36,4%	21,2%	totally agree	n=33 av.=3,6 dev.=1
The assessments (method and contents) are well matched to what you should have mastered at the end of the course.	totally disagree	0%	0%	28,1%	34,4%	37,5%	totally agree	n=32 av.=4,1 dev.=0,8
The time period set for completing the test was sufficient.	totally disagree	0%	9,1%	12,1%	42,4%	36,4%	totally agree	n=33 av.=4,1 dev.=0,9

Other -

Question	Left pole	1	2	3	4	5	Right pole	Statistics
The ratio theory/practicals was good	disagree	0%	3%	6,1%	33,3%	57,6%	agree	n=33 av.=4,5 dev.=0,8

There were sufficient assistants

disagree	1	2	3	4	5	agree	
	0%	0%	3%	27,3%	69,7%		n=33 av.=4,7 dev.=0,5

The assistants performed well

disagree	1	2	3	4	5	agree	
	0%	0%	9,1%	21,2%	69,7%		n=33 av.=4,6 dev.=0,7

There was sufficient time for writing reports

disagree	1	2	3	4	5	agree	
	0%	21,2%	15,2%	24,2%	39,4%		n=33 av.=3,8 dev.=1,2

There was sufficient time for oral presentations

disagree	1	2	3	4	5	agree	
	3%	15,2%	9,1%	27,3%	45,5%		n=33 av.=4 dev.=1,2

The method of assessment was good

disagree	1	2	3	4	5	agree	
	3,1%	0%	15,6%	43,8%	37,5%		n=32 av.=4,1 dev.=0,9 ab.=1

I have learned a lot during the practicals

disagree	1	2	3	4	5	agree	
	0%	3%	9,1%	27,3%	60,6%		n=33 av.=4,5 dev.=0,8

Online teaching -

Online teaching was satisfactory

totally disagree	1	2	3	4	5	totally agree	
	3%	3%	21,2%	60,6%	12,1%		n=33 av.=3,8 dev.=0,8

It was technically easy to follow online teaching

totally disagree	1	2	3	4	5	totally agree	
	6,1%	9,1%	12,1%	39,4%	33,3%		n=33 av.=3,8 dev.=1,2

The online exam went well

totally disagree	1	2	3	4	5	totally agree	
	0%	0%	0%	40%	60%		n=5 av.=4,6 dev.=0,5 ab.=28

There was enough interaction with the teachers

totally disagree	1	2	3	4	5	totally agree	
	0%	0%	3%	39,4%	57,6%		n=33 av.=4,5 dev.=0,6

There was enough interaction with fellow students

totally disagree	1	2	3	4	5	totally agree	
	0%	3%	9,1%	24,2%	63,6%		n=33 av.=4,5 dev.=0,8

Lecturer Martina Vijver -

The lecturer explained the subject matter well

totally disagree	1	2	3	4	5	totally agree	
	0%	0%	3%	42,4%	54,5%		n=33 av.=4,5 dev.=0,6

The presentation of lectures was inspirational

totally disagree

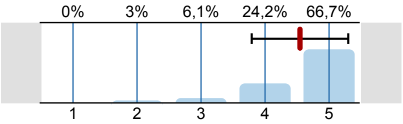

totally agree

n=33
av.=4,5
dev.=0,8

What is your **overall opinion** about this lecturer?

very poor

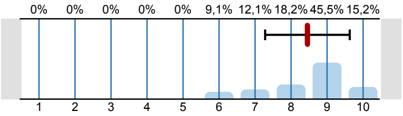

excellent

n=33
av.=8,5
dev.=1,2

Lecturer Sofie Rasmussen -

The lecturer explained the subject matter well

totally disagree

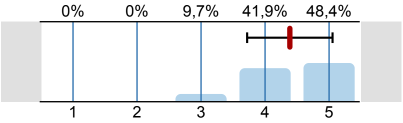

totally agree

n=31
av.=4,4
dev.=0,7
ab.=2

The presentation of lectures was inspirational

totally disagree

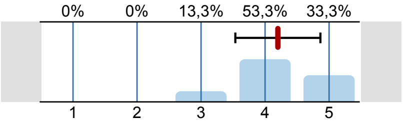

totally agree

n=30
av.=4,2
dev.=0,7
ab.=3

What is your **overall opinion** about this lecturer?

very poor

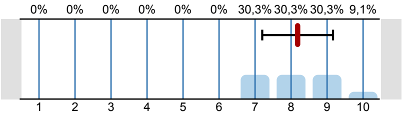

excellent

n=33
av.=8,2
dev.=1

Het EcoTox team -

The lecturers of the EcoTox team explained the subject matter well

totally disagree

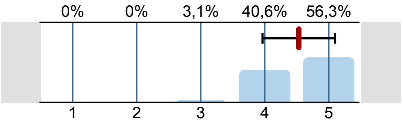

totally agree

n=32
av.=4,5
dev.=0,6
ab.=1

The presentation of lectures was inspirational

totally disagree

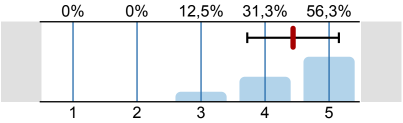

totally agree

n=32
av.=4,4
dev.=0,7
ab.=1

What is your **overall opinion** about the lecturers of the EcoTox team?

very poor

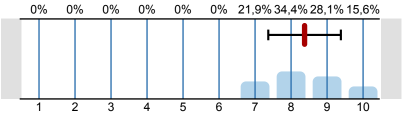

excellent

n=32
av.=8,4
dev.=1
ab.=1

Profile

Subunit: FWN master Biology IBL 21-22 [07.21.02]
Responsible for modules: Ecotoxicology - 4313ETX19
Name of the course: (Name of the survey) Ecotoxicology

Values used in the profile line: Mean

Course specific

Question	Left pole	Right pole	n	av.	md	dev.
My **overall rating** of this course on a scale from 1 to 10 (Dutch grading scale) is:	1	10	n=33	av.=8,2	md=8,0	dev.=0,9
In terms of difficulty, the course is:	too easy	too difficult	n=33	av.=3,2	md=3,0	dev.=0,5
Compared to the prescribed study load (1 EC = 28 hours including contact hours), the actual study load of this course is:	too light	too heavy	n=33	av.=3,4	md=3,0	dev.=0,7
The lecturer(s)/instructor(s) in this course teach(es) well.	totally disagree	totally agree	n=33	av.=4,4	md=4,0	dev.=0,6
The instructional methods (lecture, seminar, practical/lab work, etc.) are well matched to what you should have mastered at the end of the course.	totally disagree	totally agree	n=33	av.=4,3	md=4,0	dev.=0,8
The provision of information for this course was good.	totally disagree	totally agree	n=33	av.=4,1	md=4,0	dev.=0,9
I could easily get an answer to my questions outside of normal contact hours.	totally disagree	totally agree	n=33	av.=4,5	md=5,0	dev.=0,7
The course had the right structure/organisation to enable me to master its contents.	totally disagree	totally agree	n=33	av.=4,0	md=4,0	dev.=0,9
This course stimulates me to pursue my studies.	totally disagree	totally agree	n=33	av.=4,4	md=5,0	dev.=0,8
The online parts of this course provide effective support for my learning process.	totally disagree	totally agree	n=32	av.=4,0	md=4,0	dev.=1,0

Study material

Question	Left pole	Right pole	n	av.	md	dev.
The course materials (literature, video, podcast, Brightspace, etc.) are well matched to what you should have mastered at the end of the course.	totally disagree	totally agree	n=33	av.=4,1	md=4,0	dev.=0,8
The quality of the study material was good.	totally disagree	totally agree	n=33	av.=4,0	md=4,0	dev.=0,7
The study material on Brightspace or other digital learning environment was easy to access.	totally disagree	totally agree	n=33	av.=4,3	md=4,0	dev.=0,8
There was sufficient practice material available.	totally disagree	totally agree	n=33	av.=3,5	md=3,0	dev.=1,1

Testing

Question	Left pole	Right pole	n	av.	md	dev.
I knew in good time what I had to master for a test.	totally disagree	totally agree	n=33	av.=3,6	md=4,0	dev.=1,0
The assessments (method and contents) are well matched to what you should have mastered at the end of the course.	totally disagree	totally agree	n=32	av.=4,1	md=4,0	dev.=0,8
The time period set for completing the test was sufficient.	totally disagree	totally agree	n=33	av.=4,1	md=4,0	dev.=0,9

Other -

The ratio theory/practicals was good	disagree	agree	n=33	av.=4,5	md=5,0	dev.=0,8
There were sufficient assistants	disagree	agree	n=33	av.=4,7	md=5,0	dev.=0,5
The assistants performed well	disagree	agree	n=33	av.=4,6	md=5,0	dev.=0,7
There was sufficient time for writing reports	disagree	agree	n=33	av.=3,8	md=4,0	dev.=1,2
There was sufficient time for oral presentations	disagree	agree	n=33	av.=4,0	md=4,0	dev.=1,2
The method of assessment was good	disagree	agree	n=32	av.=4,1	md=4,0	dev.=0,9
I have learned a lot during the practicals	disagree	agree	n=33	av.=4,5	md=5,0	dev.=0,8

Online teaching -

Online teaching was satisfactory	totally disagree	totally agree	n=33	av.=3,8	md=4,0	dev.=0,8
It was technically easy to follow online teaching	totally disagree	totally agree	n=33	av.=3,8	md=4,0	dev.=1,2
The online exam went well	totally disagree	totally agree	n=5	av.=4,6	md=5,0	dev.=0,5
There was enough interaction with the teachers	totally disagree	totally agree	n=33	av.=4,5	md=5,0	dev.=0,6
There was enough interaction with fellow students	totally disagree	totally agree	n=33	av.=4,5	md=5,0	dev.=0,8

Lecturer Martina Vijver -

The lecturer explained the subject matter well	totally disagree	totally agree	n=33	av.=4,5	md=5,0	dev.=0,6
The presentation of lectures was inspirational	totally disagree	totally agree	n=33	av.=4,5	md=5,0	dev.=0,8
What is your **overall opinion** about this lecturer?	very poor	excellent	n=33	av.=8,5	md=9,0	dev.=1,2

Lecturer Sofie Rasmussen -

The lecturer explained the subject matter well	totally disagree	totally agree	n=31	av.=4,4	md=4,0	dev.=0,7
The presentation of lectures was inspirational	totally disagree	totally agree	n=30	av.=4,2	md=4,0	dev.=0,7
What is your **overall opinion** about this lecturer?	very poor	excellent	n=33	av.=8,2	md=8,0	dev.=1,0

Het EcoTox team -

The lecturers of the EcoTox team explained the subject matter well	totally disagree	totally agree	n=32	av.=4,5	md=5,0	dev.=0,6
The presentation of lectures was inspirational	totally disagree	totally agree	n=32	av.=4,4	md=5,0	dev.=0,7
What is your **overall opinion** about the lecturers of the EcoTox team?	very poor	excellent	n=32	av.=8,4	md=8,0	dev.=1,0

Comments Report

General -

Other programme, namely:

The evaluation will not be displayed due to low response rate.

Remarks about this subject / explanation of your answers to the previous questions -

Strong points are:

- A lot of enthousiasm!
- All lecturers were very passionate about the topics which was inspiring. Also nice to do some real lab work and interesting that we touched upon many topics
- All the lectures were given very insparational and enthousiastic!
- Course triggers further study and interest for students due to the enthousiasm of all teachers. Also the team really supported us during practicals and lectures, which made learning nice and pleasent.
  Also a very strong point: good structure of lectures in first week, with very useful tools and knowledge for not only this course, but also for many other future careers, jobs, positions.
- Getting to work in the lab with other students
- Good content, inspiring lectures and practicals.
- Good elaboration on critical thinking
  Active discussion
  Responsive staffs
  Nice practical study
- Good interaction with students, the lecturer and assistants. Both lecturer and assistants are enthusiastic and eager to help.
- Good way of interactions with fellow students, good help and interesting lectures of all speakers, inspiring subjects and most of all, learned a lot of both theoretical knowledgse and practical skills.
  Liked the way of teamwork, combining strengths of several students.
  Nice wrap up lecture on Ecotoxicology management. Thanks for all effort, really happy to attend this course.
- I loved the lab work! Really missed doing some hands-on practicals & it felt like it was meaningful too!
- I really liked the course and learned a lot. It was completely different from previous courses thusfar in a very good way.
  I loved the fact that it was finally a practical course in stead of a complete theoretical course and that we were allowed to work in the lab.
- I think a strong points about the course is the combination of practical work and lectures. It is very nice to see the information in the practical world.
- It is very interesting and the enthausiasm of the entire team was inspirational
- Martina is an amazing researcher who teaches very enthusiastically. She also did her best to get to know the students and talked a lot with us. The rest of the team did the same. At first I was a little sceptic about how much I would like the course content but Martina totally changed my mind.
- Martina's limitless enthusiasm
- Onwijs leuk om onderwijs te krijgen van zulke enthousiaste docenten, met veel interactie en practica
- Passionate lecturers. Practicals were very nice. The freedom we got was really good. We had to think for ourselves and “just try” which was really nice. But we also got good help when we needed it.
- Practicals
  Enthousiams of the ecotox team
- Thank you for a very interesting course. I learned a lot of new things and the material and topics were very relevant.
- The ecotox team is a group of inspiring experts that give interesting lectures.
  I really liked the practical part of this course, I finally felt like a student again!
- This course give a lot of insights into the field of ecotoxicology, but also research in general. It invites you to really think about what you are doing, what your results mean, and what this means in the bigger picture.
  The teachers were very good, inviting you to open discourse about the subjects and really inspiring you!
- This was seriously a perfect master course. First, the content was very interesting a

And the structure was very efficient and advantegous. That the first week was meant for prepertation and backround knowledge, and setting up groups and ideas, worked very well. Also the part of statistical power is very useful not only for this course, but for every future career task. Also all teachers were helpfull and inspiring during lectures, both of these things really help to work out things quickly. Together, I strongly believe this course was valuable/useful to gain ecotoxilogical knowledge and also scientific skills in general. Finally, loved the way we worked in groups in the labs. This way you learn a lot, by learning from others and put all knowledge in practice. Thanks for everything.

- With the practicals and lectures from PhD students, you really learned how ecotox studies are performed and what you could expect when you work in this field
- Working in the lab/ lots of practical experience
  Seeing people in real life
- communication via Teams
  enthusiasm
  opportunity for creativity
- online book exam which makes you think instead of just study a lot of information you could otherwise look up. Martina is a fun teacher who looks at the course material from different angles and really lets you think.
- the practical part of the course is really nice because you really learn what it is like to be a scientist in action.

Weak points are (please suggest improvements):

- - Scheduled breaks and sticking to them. Especially online. It is very hard to study online, the breaks are really really important. A break of 5 min every 45 min is the minimum! Lectures of 1:15-1:45h are not possible with full concentration.

  It was quite hard to stay within the word limit, even if we know it Should be a relief :). Maybe a higher limit (eg 4000) would be better for a group of 6p
- A little chaotic start of experiments, that's why we had too lilttle time for every 0-measurement at Day 1. Also not clear where lectures where given, sometimes location of time was changed without an announcement.
  For the rest, it was perfectly organized and very well supported by everyone of the Ecotoxteam.
- Can't think of any.
  I skipped some online lectures to do some labwork but there was enough time in the end to catch up with them.
- Het statistiek element (power analyse) was wel nuttig voor de practica maar verder had ik het meeste al gezien tijdens advanced statistics, dus misschien kan het daar wat meer op worden afgesteld.
- I know this cource is only thought every 2 years, but maybe some better preperation of online tools or scedules would be nice.

  The scedule in the first week was really badly organised, without clear breaks etc, and this wasnt necesarry.
  Also the online streaming wasnt that good and you felt a bit unheard if you wanted to say something about it
- I missed more theoretical knowledge on toxicokinetics and -dynamics and it would have helped to create an in dept understanding of how toxins end up in the organism and which factors play a role in it. Now it was only covered by the lecture on bioavailability and I would have preferred if there were more lectures like that
- I think it would be good to set some guidelines for all lab asistants as there were some contradicting answers between some of the different assistants when we asked questions. It did work out at the end though.

  Also I would have liked to have a bit more time for writing a good paper, with a lecture everyday it was not really doable to get going with that, which gave us some time pressure and long weekend work on doing the analysis which is not as good as it could have been. Maybe try to really push students to start thinking on how they will analyze their data earlier on in the course
- I would have like some mor depht in some cases like hoe is it toxic which proteins does a substance interact with. But that could also be because i followed a study path in toxicologie in my bachalor so many thing that were taught in the course I already knew.
- It was not clear at the beginning if a course was going to be online or offline. This resulted in a big group expecting the lecture to be offline while it was online. We had to wait an hour for it to start, in which we all could have been home. The week after this went better.
  The whole course focusses on an essay and the laboratory work that is needed for that paper. The fact that there is a test, just feels weird. However, the way this exam was structured, did make sense.
  The time scheduled for the lectures was almost never met. This way it is hard to make a planning of what we wanted to do when during the laboratory work. You never knew how long the lecture would eventually take. Another thing about the lectures is the lack of breaks. Breaks are highly necessary and should be there for sure. I really struggled with this fact and trying to keep paying attention even though it was already going for one and a half hour.
  The presentations on Monday with the statistical analysis is not doable. The weekend is not ment to work on school and most students have work, matches, meeting with friends etc. To expect us to finish work on Friday and then to present the findings on Monday is not realistic.
  Part of the course and materials was on teams, on different pages, and part was on Brightspace, again on different pages. It would be better to have at least all lecture slides and other materials on Brightspace. That way we know where to look for materials. Right now it was hard to find some of the lectures and I'm not even sure if all was made available.
- None.
- Not clear what material was available for practical, somethimes contardicting opinions of teachers. But not a big problem of course, this also happens in real life.

- Sometimes a little too passionate. Also we had little time to write report and make presentations. Maybe because we had a lot more data to process than other groups.

- The first days of lectures were very hard to keep up with. It´s near impossible to keep concentration with 4 hours of online lectures with little breaks. All other days were fine though!

- The first week of online lectures was a little bit exhausting due to fewer breaks.

- The first week was online, which was unavoidable. However, during online teaching, Martina tended to forget breaks during lectures. We would have lectures for 3 hours straight, then a break of 10-15 minutes, and then another few hours. This does not change anything about my opinion of Martina!

- The lectures, especially in the first week, were too long and not according to the schedule
  The communication to the students about practical matter was not always optimal. Maybe make a brightspace announcement instead of an oral announcement or in MSTeams
  The time to do the statistical analysis before the presentation was too short

- The start of the course was quite densely packed and I felt we could have used a bit more time to familiarise ourselves with the lab and with the recommended computer programmes. As it is the start of the practical seemed a bit rushed, leading to a bit of uncertainty and perhaps impacting the overall quality of the practical.

- The time frame to come up with a research question, design an experiment and present it was too short and pressuring. Students may have more time (at least a week) for this process or they can be informed more early on for what is needed. Especially the first week the classes were too long and difficult to follow, the breaks were not structured well. Also the lectures took more than planned multiple times (30-45 min) which interferes with the students` plans. The lectures can be structured better.

- The time we had for the start of the assignment was very short.

- Too early morning for exam, 10.00 should be more comfortable especialy for those who need to travel

- Very positive about this course. The only minor weak point was in the first week, that there were no breaks scheduled (or duration of lectures went over time) so we had to drink or eat quickly behind the computer. Not a big deal but it can be imporved next time.

- Weak points:
  - It would be very nice for online lectures to stay within the preannounced timeframes. This is what the students expected. It is very hard to stay focused when the lecturer keeps going on for an unlimited amount of time without any breaks. It is very nice to know when you have a break and how long you have to have the focus.

  - I think once we switched to hybrid lectures it became a lot harder for people to follow it online. I get that it is a more stressful to also keep in mind to start this up. However the first lectures was therefore so hard to follow with very small screens which made it almost impossible to follow in a decent way. Martina didn't want to fix it and I almost got the feeling we just had some bad luck and if we wanted to follow the lecture we just had to be there in real life.
  In my bacherlor (utrecht) every lecture was standard recorded (pre corona) which had a very positive effect on the students. Almost all students came to the lectures but it is super efficient to watch certain parts back which where difficult to follow or just a difficult subject. What I want to say is that it is not so negative to have to record the lectures. It is almost always very much appreciated.

- safety concerns in the lab -> providing glasses, more supervision
  hybrid teaching -> when online it was often hard to follow the in-person lecture (people were often home because of covid reasons so really dependent on online lectures)

- there were 2 weeks of practical work for which you were assigned in a group of 7 people. This resulted in a very low workload and depending on how the work was divided, you didnt get a lot of actual lab work done. So for me this was a pity and it also resulted in that it didnt really feel as an 6 ECT master course. I think smaller practical work groups would maybe work better.
