# Supplementary material for: Participatory hackathon to determine ecological relevant endpoints for a neurotoxin to aquatic and benthic invertebrates
Source: Environ Sci Pollut Res Int. 2024 Feb 28;31(15):22885–99. doi: 10.1007/s11356-024-32566-w (PMC10997722; doi:10.1007/s11356-024-32566-w)
Supplement: Supplementary file 3 — (DOCX 284 kb) [file 11356_2024_32566_MOESM3_ESM.docx]

**Participatory Hackathon to determine ecological relevant endpoints for a neurotoxin to aquatic and benthic invertebrates**

Sofie B. Rasmussen^1, *^, Thijs Bosker ^1,2^, Giovani G. Ramanand^1^, Martina G. Vijver^1^

^1^ Institute of Environmental Sciences, Leiden University, P.O. Box 9518, 2300 RA Leiden, the Netherlands

^2^ Leiden University College, Leiden University, P.O. Box 13228, 2501 EE, The Hague, the Netherlands

^*^Corresponding author, Institute of Environmental Sciences, Leiden University, P.O. Box 9518, 2300 RA Leiden, the Netherlands. Email: [a.s.b.rasmussen@cml.leidenuniv.nl](mailto:a.s.b.rasmussen@cml.leidenuniv.nl), tel.: +45 20334344

**For submission in Environmental Science and Pollution Research**

**Dose response curves of *C. riparius* exposed to sulfoxaflor**

***Figure 1*** *Dose-response relationship is based on actual concentrations. Effect level are mean difference from control group and model is fitted to two parameters; min=0 and max=1, for binomial responses using a log-logistic function. The rest is fitted using a four-parameter log-logistic function. Confidence intervals are portrayed in grey. a) Effect on survival of C. riparius larvae at Day 9 (n=12). b) Effects on activity measured as fleeing response in sediment of C. riparius larvae at Day 3 (n=8). c) Effect on growth of C. riparius larvae at Day 9 (n=10). d) Effects on mean velocity in water phase of C. riparius, measured using video tracking larvae over a period of 10 minutes (n=12). e) Effects on emergence of adults (n=10). f) Effects on duration of time in 10 minutes each larvae spend swimming at Day 9 (n=12). g) Effects on emergence of adults per surviving larvae at Day 9 (n=10). h) Effects on duration of time in 10 minutes each larvae spend not moving at Day 9 (n=12).*
