# Supplementary material for: Participatory hackathon to determine ecological relevant endpoints for a neurotoxin to aquatic and benthic invertebrates
Source: Environ Sci Pollut Res Int. 2024 Feb 28;31(15):22885–99. doi: 10.1007/s11356-024-32566-w (PMC10997722; doi:10.1007/s11356-024-32566-w)
Supplement: Supplementary file 6 — (DOCX 21 kb) [file 11356_2024_32566_MOESM6_ESM.docx]

**Participatory Hackathon to determine ecological relevant endpoints for a neurotoxin to aquatic and benthic invertebrates**

Sofie B. Rasmussen^1, *^, Thijs Bosker ^1,2^, Giovani G. Ramanand^1^, Martina G. Vijver^1^

^1^ Institute of Environmental Sciences, Leiden University, P.O. Box 9518, 2300 RA Leiden, the Netherlands

^2^ Leiden University College, Leiden University, P.O. Box 13228, 2501 EE, The Hague, the Netherlands

^*^Corresponding author, Institute of Environmental Sciences, Leiden University, P.O. Box 9518, 2300 RA Leiden, the Netherlands. Email: [a.s.b.rasmussen@cml.leidenuniv.nl](mailto:a.s.b.rasmussen@cml.leidenuniv.nl), tel.: +45 20334344

**For submission in Environmental Science and Pollution Research**

Method on chemical analysis: Sulfoxaflor

Equipment

- UPLC; Waters Aquity I-class FTN
- Column; Waters ACQUITY UPLC BEH C18（2.1mm×50 mm, 1.7 μm）
- Formic Acid
- HPLC water
- HPLC Acetonitril
- Sulfoxaflor standard 98% purity; can be purchased from Toronto Research Chemicals Inc.
- Sulfoxaflor-d3 internal standard; can be purchased from Toronto Research Chemicals Inc.
- Mass spec system; ScieX Qtrap 6500
- 1,5 ml vials

Method

To determine Sulfoxaflor concentrations, an UPLC, Waters Aquity I-class FTN was coupled to a Mass spec system, ScieX Qtrao 6500. The column used was a Waters ACQUITY UPLC BEH C18 2.1mm×50 mm, 1.7 μm). Two eluents were used: eluant A (95% H2O, 5% ACN, 0.1% Formic acid) and eluant B (95% ACN, 5% H2O, 0.1% Formic acid). The gradient can be seen in Table 1. The source and system settings for the MS can be seen in Table 2. Multi-reaction monitoring in positive ion mode (MRM) was selected as the scan mode. The settings for the MRM mode can be seen in Table 3. A sulfoxaflor standard was used to make stock solutions. From these stock solutions, a calibration with a range of 0 ppb – 200 ppb sulfoxaflor was made. All the samples were transferred in 1..5 ml sample vials. The sample volume was 1 ml. The internal standard was added to all the sample vials. The final concentration of the internal standard was 10 ppb. All the samples were measured three times.

Table 1: Gradient UPLC

| Min. | Flow (Ml/Min) | % Eluent A | % Eluent B |
| --- | --- | --- | --- |
| 0,00 | 0,60 | 98 | 2 |
| 0,20 | 0,60 | 98 | 2 |
| 2,00 | 0,60 | 20 | 80 |
| 4,00 | 0,60 | 20 | 80 |
| 4,10 | 0,60 | 98 | 2 |
| 6,00 | 0,60 | 98 | 2 |

Table 2: Source and systems settings MS

| Ion Source | Turbo Spray |
| --- | --- |
| Curtain Gas | 40 |
| Collision Gas | Medium |
| Ion Spray Voltage | 5500 |
| Temperature | 200 |
| Ion Source Gas 1 | 60 |
| Ion Source Gas 2 | 60 |
| Declustering Potential | 10 |
| Entrance Potential | 10 |
| Collision Energy | 30 |
| Collision Cell Exit Potential | 10 |

Table 3: Settings MRM mode

| Precursor | Product | Time (ms) | ID |
| --- | --- | --- | --- |
| 278 | 174 | 50 | Sulfoxaflor |
| 278 | 154 | 50 | Sulfoxaflor |
| 281 | 177 | 50 | Sulfoxaflor-d3 |
| 281 | 156 | 50 | Sulfoxaflor-d3 |
| 295 | 174 | 50 | Sulfoxaflor (NH3) |
| 295 | 154 | 50 | Sulfoxaflor (NH3) |
